# Rapid diagnostics of orthopedic implant-associated infections using Unyvero ITI implant and tissue infection application is not optimal for *Staphylococcus* species identification

**DOI:** 10.1186/s13104-019-4755-5

**Published:** 2019-11-06

**Authors:** Hege Vangstein Aamot, Bjørn Odd Johnsen, Inge Skråmm

**Affiliations:** 1 Department of Microbiology and Infection Control, Akershus University Hospital, Lørenskog, Norway; 2 Department of Clinical Molecular Biology (Epigen), Akershus University Hospital and University of Oslo, Lørenskog, Norway; 3 Department of Orthopedic Surgery, Akershus University Hospital, Lørenskog, Norway; 4 Present Address: Department of Medicine, Vestre Viken Hospital Trust, Ringerike Hospital, Hønefoss, Norway

**Keywords:** Rapid diagnostics, Implant infection, *Staphylococcus aureus*, Unyvero ITI, Orthopedic

## Abstract

**Objectives:**

This pilot study aimed to compare the commercial Unyvero ITI multiplex PCR application (U-ITI, Curetis GmbH) with conventional culturing concerning (a) detection of pathogens, (b) time to detection of pathogens and (c) time to and quality of antibiotic treatment recommendation in diagnostics of orthopedic implant-associated infections (OIAI).

**Results:**

72 tissue biopsies from 15 consecutive patients with deep OIAI infections were analyzed with conventional culturing including phenotypic antibiotic susceptibility testing and the U-ITI. U-ITI showed lower sensitivity than conventional culturing concerning detection of pathogens (73% vs 93%). 4/15 patients would have been given false negative results by U-ITI, all of which were culture-positive for *Staphylococcus* species. Median time to detection of pathogens was 47 h and antibiotic resistance 89 h by conventional methods compared to 13.5 h with the U-ITI. The U-ITI did not detect antibiotic resistance, whereas conventional culturing showed resistance to antibiotics covered by the U-ITI panel in 2 patients. Time to detection of pathogens was improved, but the detection limit for staphylococci was unsatisfactory. Although the time to antibiotic treatment recommendation was significantly reduced, the U-ITI would have resulted in incorrect antibiotic recommendation in 2 patients. Our data do not support use of this assay in diagnostics.

## Introduction

The majority of orthopedic procedures involve the use of implants. Implants dramatically increase the risk of infection [1]. Although these infections are infrequent, with an overall surgical site infection rate following implant surgery of 3% [2], the number of patients undergoing orthopedic implant surgery increases.

Current standard procedure for identification of the microbes causing these infections is extensive [3] and only few diagnostic tools for rapid diagnostics of orthopedic implant-associated infections (OIAI) with varying degrees of sensitivity and specificity are available [4, 5]. However, the commercial Unyvero ITI multiplex-PCR application (U-ITI) for identification of implant and tissue infections (Curetis GmbH, Holzgerlingen, Germany) can detect selected pathogens and antibiotic resistance markers within a few hours analyzing more than 100 DNA targets simultaneously. This allows for more expeditious microbe identification and administration of targeted treatment than conventional culturing.

This study aims to compare the U-ITI assay to conventional culturing in diagnosing orthopedic implant-associated infections (OIAI). The parameters compared include (a) detection of pathogens, (b) time to detection of pathogens, and (c) time to and quality of antibiotic treatment recommendations.

## Main text

### Materials and methods

Patients with acute, clinically defined deep OIAIs necessitating revision surgery from January through August 2017 at Akershus University Hospital, Lørenskog, Norway were eligible for inclusion. The criteria for an OIAI were based on the standards described by Parvizi [6] with a clinically motivated adjustment for patient ID 101.

Diagnostic soft tissue biopsies were routinely collected intraoperatively. According to international consensus, 5 biopsies should be collected [3]. If more than 5 tissue biopsies were collected, 5 biopsies were selected at random for Unyvero analysis. In cases with less than 5 biopsies, all were included. No patients received antibiotics prior to surgery, except for patient ID 101 who received penicillin due to a skin infection. According to conventional guidelines, empirical treatment were started after biopsies were taken.

All biopsies were cut into three: one followed standard culture procedure, one was analyzed with Unyvero, and the last was stored at − 80 °C. If it was not possible to perform the Unyvero analysis within 48 h of surgery, the biopsies were stored at − 80 °C. Otherwise, the biopsies were analyzed consecutively and temporarily stored at 4 °C.

Standard culturing was performed by homogenizing the sample with mortar and pistil in Mueller–Hinton broth in a type 2 microbiological safety cabinet with subsequent seeding, using a modified quadrant streak technique with only 3 “quadrants”, of:

1. 1 blood agar plate (incubation 5 days aerobically at 35 °C with a regular atmosphere supplemented with 5% CO_2_).
2. 1 chocolate agar plate incubated aerobically as described for 5 days.
3. 1 plate of tryptose soy agar base supplemented with 5% defibrinated sheep blood, 0.001% vitamin K, 0.0005% hemin, 0.1% glucose and 0.03% yeast extract (incubation 5 days in anaerobic chamber at 35 °C with an atmosphere containing 10% CO_2_, 10% H_2_ and 80% N_2_),
4. 1 Mueller–Hinton broth (incubation aerobically for 2 days before subcultivation to a blood agar plate and a chocolate agar plate incubated aerobically for another 3 days).

All agars and broths were manufactured by the department’s own media production unit. Bacterial growth was semiquantified using the designations sparse, moderate or rich growth. The colonies were subcultured in the relevant atmosphere and identified by matrix assisted laser desorption ionization time of flight (MALDI-TOF) using MALDI-TOF MS Biotyper (Bruker Daltonik GmbH, Bremen, Germany, MBT 6903 MSP Library, MBT Compass v4.1.70.1, Compass for flexControl v3.4). A specific bacterium had to be growing in at least 2 tissue biopsies or be detected by U-ITI in at least 2 tissue biopsies per patient to be considered positive. Antibiotic susceptibility testing was performed according to the guideline from the European Committee on Antimicrobial Susceptibility Testing EUCAST [7] and EUCAST breakpoints were utilized to categorize the isolate as sensitive (S), intermediate (I) or resistant (R) [8].

Time to pathogen detection was defined as the time from sampling the tissue biopsy to the time of pathogen identification. Similarly, time to antibiotic treatment recommendation and complete results, were defined as the time from sampling of tissue biopsy to time of reporting the results of antibiotic susceptibility testing and all other results, including anaerobic cultivation.

The Unyvero U-ITI assay consists of a sample tube with pre-treatment buffer, a sealed master mix tube and a cartridge where the multiplex PCR is performed. The results are reported as positive or negative for each microbe/resistance marker and the degree of positivity is reported as 1–3 green boxes. Unyvero analysis was performed on the Unyvero system, consisting of a lysator, analyser and cockpit, as recommended by the manufacturer (Curetis GmbH, Holzgerlingen, Germany). The analyzer can perform multiplex-PCR on 2 tissue samples at a time. When analyzing 5 tissue samples, the total time from biopsy to finished results would be approximately ~ 13.5 h if analyzed consecutively.

Sensitivity of both methods was calculated as the number of patients that were positive for a pathogen in at least two biopsies divided on the total number of patients that had a clinically defined infection.

### Results

#### Detection of pathogen

72 tissue biopsies from 15 consecutive patients were included. 9 (60%) patients were females and median age was 72 years [range: 42–88 years]. The criteria met for OIAI for each patient are presented in Additional file 1. The infected implants were joint prosthesis in 10 patients, and osteosynthetic devices in 5 patients. Of the 72 biopsies, 50 were analyzed consecutively, whereas 22 biopsies were analyzed after storage in − 80 °C. The distribution of results from culture and Unyvero results is presented in Table 1. Detailed results from identification of the pathogens by the two different methods are presented in Table 2. Standard culturing methods showed higher sensitivity than the Unyvero ITI application with 62 versus 43 positive tissue biopsies (Table 1). The biopsies only positive by culturing included 18 biopsies positive for *Staphylococcus aureus* and 8 biopsies positive for *Staphylococcus epidermidis*. 4 patients suffering from OIAI with *S. aureus*, coagulase negative staphylococci or both, would not have had an aetiological diagnosis using U-ITI alone. Conversely, U-ITI identified the pathogen in 6 culture-negative tissue biopsies from two patients (Table 2, IDs 101 and 115) which were positive for *Propionibacterium acnes* and/or *Streptococcus pneumoniae*. In addition, U-ITI identified two additional bacteria from one patient (coagulase negative staphylococci and *Finegoldia magna* in addition to *Corynebacterium* species, Table 2, ID102). Sensitivity on the patient level was 93% (CI 68–100%, 14/15 patients) for culturing, whereas sensitivity for Unyvero was 73% (CI 45–92%, 11/15 patients).

**Table 1:** Distribution of results of conventional culturing versus Unyvero ITI multiplex PCR of 72 biopsies from 15 patients with orthopaedic implant-associated infections

	Unyvero negative	Unyvero positive	Total
Culture negative	4 (0)	6 (1)	10 (1)
Culture positive	25^a^ (4)	37^a^ (10)	62 (14)
Total	29 (4)	43 (11)	72 (15)

The number of patients affected when the results from all biopsies are considered is given in parenthesis

^a^Including 1 invalid of a total of 8 multiplex-PCR Unyvero

**Table 2:** Identification of pathogens by conventional culturing and Unyvero ITI application of 72 tissue biopsies from 15 orthopaedic patients with orthopaedic implant-associated infections

Patient	Biopsy number	Standard culturing					Unyvero analysis		
		Time to detection of pathogens (h)	Time to recommendation antibiotic treatment (h)	Time to complete results (h)	Pathogen ID	Growth quantification	Pathogen ID	Degree of PCR positivity (1–3)	Tissue frozen prior to analysis
101	1			137.7	Neg	–	Neg	–	
101	2				Neg	–	*S. pneumoniae*	2	
							*P. acnes*	1	
101	3				Neg	–	Neg^a^	–	
101	4				Neg	–	*S. pneumoniae*	2	
101	5				Neg	–	*S. pneumoniae*	1	
102	1	65.0	88.6	186.2	*C. jeikeium*	Broth only	CNS	1	
102	2				*C. jeikeium*	Sparse	*Corynebacterium* spp.	1	
102	3				*C. jeikeium*	Broth only	*Corynebacterium* spp.	1	
102	5				*C. jeikeium*	Broth only	Neg	–	Yes
102	6				*C. jeikeium*	Broth only	*F. magna*	2	Yes
104	1	68.7	192.3	192.3	*S. lugdunensis*	Sparse	CNS	3	
104	2				*S. lugdunensis*	Sparse	CNS	2	
104	3				*S. lugdunensis*	Sparse	Neg	–	
					*P. acnes*	Sparse			
104	4				*S. lugdunensis*	Sparse	CNS	2	
104	5				*S. lugdunensis*	Sparse	Neg^a^	–	
					*P. acnes*	Sparse			
105	1	58.7	156.0	156.0	Neg	–	Neg	–	
105	2				*S. aureus*	Broth only	Neg	–	
					*S. epidermidis*	Broth only			
105	3				*S. aureus*	Sparse	Neg	–	
					*S. epidermidis*	Broth only			
					*S. capitis*	Sparse			
105	4				*S. aureus*	Broth only	Neg	–	
					*S. epidermidis*	Broth only			
105	5				*S. aureus*	Sparse	Neg	–	
					*S. epidermidis*	Broth only			
107	1	96.0	96.0	170.8	*S. epidermidis*	Sparse	Neg	–	
107	2				*S. epidermidis*	Broth only	Neg	–	
107	3				*S. epidermidis*	Sparse	Neg	–	
107	4				*S. epidermidis*	Broth only	Neg	–	
107	5				Neg	–	Neg	–	
108	1	24.3	47.8	122.8	*S. aureus*	Sparse	*S. aureus*	2	
108	2				*S. aureus*	Sparse	*S. aureus*	1	
108	3				*S. aureus*	Sparse	*S. aureus*	1	
108	4				*S. aureus*	Sparse	*S. aureus*	1	
108	5				*S. aureus*	Sparse	*S. aureus*	1	
109	1	43.1	63.6	137.1	*S. aureus*	Sparse	Neg	–	
109	2				*S. aureus*	Sparse	Neg	–	Yes
109	3				*S. aureus*	Sparse	Neg	–	
109	4				*S. aureus*	Sparse	Neg	–	
109	5				*S. aureus*	Sparse	Neg	–	
110	1			105.8	*S. aureus*	Sparse	Neg	–	
110	2				*S. aureus*	Sparse	Neg	–	
110	3				*S. aureus*	Sparse	Neg	–	
110	4				*S. aureus*	Sparse	Neg	–	
110	5				*S. aureus*	Sparse	Neg	–	
111	1	53.6	76.1	147.3	*S. aureus*	Rich	*S. aureus*	2	
111	2				*S. aureus*	Rich	*S. aureus*	2	Yes
111	4				*S. aureus*	Rich	*S. aureus*	2	
111	5				*S. aureus*	Rich	*S. aureus*	2	
111	6				*S. aureus*	Rich	*S. aureus*	2	
112	1	46.6	46.6	143.7	*S. aureus*	Sparse	*S. aureus*	2	Yes
112	2				*S. aureus*	Sparse	*S. aureus*	2	Yes
112	3				*S. aureus*	Rich	*S. aureus* ^a^	3	Yes
112	4				*S. aureus*	Sparse	*S. aureus*	2	Yes
113	1	23.5	43.9	142.9	*S. aureus*	Rich	*S. aureus*	2	Yes
113	2				*S. aureus*	Rich	*S. aureus*	2	Yes
113	3				*S. aureus*	Rich	*S. aureus*	2	Yes
113	4				*S. aureus*	Rich	*S. aureus*	2	Yes
114	1	24.3	46.1	120.2	*S. aureus*	Rich	*S. aureus*	2	Yes
114	2				*S. aureus*	Moderate	*S. aureus*	2	Yes
114	3				*S. aureus*	Rich	*S. aureus*	3	Yes
114	4				*S. aureus*	Moderate	*S. aureus*	2	Yes
114	5				*S. aureus*	Moderate	*S. aureus*	2	Yes
115	1			167.7	Neg	–	*P. acnes*	2	Yes
115	2				Neg	–	*P. acnes*	1	Yes
115	3				Neg	–	*P. acnes*	2	Yes
115	4				*P. acnes*	Sparse	*P. acnes*	3	Yes
115	5				*P. acnes*	Sparse	*P. acnes*	1	Yes
116	1	26.7	69.3	119.2	*S. aureus*	Sparse	Neg	–	
116	2				*S. aureus*	Sparse	*S. aureus*	1	
116	3				*S. aureus*	Sparse	*S. aureus*	1	
116	4				*S. aureus*	Sparse	Neg	–	
116	5				*S. aureus*	Sparse	*S. aureus*	1	
117	1	20.3	115.7	115.7	*S. aureus*	Moderate	*S. aureus*	1	
117	2				*S. aureus*	Sparse	Neg	–	
117	3				*S. aureus*	Sparse	*S. aureus*	1	
117	4				*S. aureus*	Sparse	Neg	–	

^a^One PCR chamber invalid

*Neg* negative, *CNS* coagulase negative staphylocci, *spp.* species

#### Response times

Median time to detection of pathogen by conventional culturing was 47 h [range: 20–168 h], whereas median time to results from antibiotic sensitivity was 89 h [range: 44–192 h]. Median time to final results, including results from anaerobic culturing, was 143 h [range: 106–192 h] by conventional methods. The corresponding analysis time for U-ITI would be a maximum of 13.5 h if analyzed consecutively.

#### Quality of antibiotic treatment advice

Phenotypic identification of antibiotic resistance and its correlation with genes detectable by U-ITI are presented in Table 3. U-ITI identified no resistance genes. Conventional phenotypic testing was able to detect resistance to several antibiotics, however, none of the antibiotic resistance phenotypes detected here are among those detectable by the U-ITI. Additionally, in a total of 4 biopsies from 2 patients U-ITI gave false negative results.

**Table 3:** Antibiotic resistance: phenotypic identification with conventional methods versus genotypic identification with Unyvero ITI application of 72 tissue biopsies from 15 patients with orthopaedic implant-associated infection

Standard phenotypic identification			Possible genotypic identification—Unyvero^b,c^	Relevant for the following species detected in this pilot
Antibiotics tested	N resistant microbes/N microbes tested^a^	Patient ID of patients with resistant microbes		
Cefoxitin (methicillin resistance screening)	1/13	107	mecA, mecC	*Staphylococcus* spp.
Ciprofloxacin	2/13	107, 117	–	*Corynebacterium* spp.
				*Staphylococcus* spp
Erythromycin	1/13	107	ermA, ermC	*Staphylococcus* spp.
				*Streptococcus pneumoniae*
Fusidic acid	3/13	105 × 2, 107	–	*Staphylococcus* spp.
Gentamicin	0/14		aa(6′)aph(2″), aacA4	*Corynebacterium* spp.
				*Staphylococcus* spp.
Clindamycin	2/15	102, 107	ermA, ermC	All
Chloramphenicol	0/9		–	*Finegoldia magna*
				*Propionibacterium acnes*
				*Staphylococcus* spp.
				*Streptococcus pneumoniae*
Linezolid	0/13		–	*Corynebacterium* spp.
				*Staphylococcus* spp.
				*Streptococcus pneumoniae*
Meropenem^c^	1/14^c^		kpc, imp, ndm, oxa-23, oxa-24/40, oxa-48, oxa-58, vim	*Finegoldia magna*
				*Propionibacterium acnes*
				*Staphylococcus* spp.^c^
				*Streptococcus pneumoniae*
Metronidazole	1/1	104	–	*Finegoldia magna*
Penicillin	11/14	102, 104, 105 × 2, 108, 109, 111, 113, 114, 116, 117	–	All
Piperacillin Tazobactam ^c^	1/14^c^		–	*Finegoldia magna*
				*Propionibacterium acnes*
				*Staphylococcus* spp.^c^
				*Streptococcus pneumoniae*
Rifampicin	0/13		–	*Corynebacterium* spp.
				*Staphylococcus* spp.
				*Streptococcus pneumoniae*
Teicoplanin	0/4		vanA	*Finegoldia magna*
				*Propionibacterium acnes*
				*Staphylococcus* spp.
				*Streptococcus pneumoniae*
Tetracycline	0/12		–	*Corynebacterium* spp.
				*Staphylococcus* spp.
				*Streptococcus pneumoniae*
Trimethoprim–sulfamethoxazole	1/13	107	–	*Staphylococcus* spp.
				*Streptococcus pneumoniae*
Vancomycin	0/4		vanA, vanB	All
3rd generation cephalosporins^d^	1/13^c^		CTX-M	*Staphylococcus* spp.^d^
				*Streptococcus pneumoniae*

^a^Patient 115 failed phenotypic antibiotic resistance testing

^b^Underline types indicate genes relevant for bacteria detected in this pilot

^c^All biopsies were negative

^d^Phenotypically cefoxitin sensitive/genotypically mecA and mecC negative staphylococci are considered sensitive to meropenem, piperacillin–tazobactam and 3rd generation cephalosporins active against staphylococci

### Discussion

The U-ITI was inadequate in rapid identification of bacteria and antibiotic resistance. The sensitivity was 73% for U-ITI compared to a sensitivity of 93% for conventional culturing. The inadequacy was particularly evident in the detection of *S. aureus* as 18 biopsies positive for *S. aureus* by standard culturing were negative by the U-ITI (Table 2). Additionally, 8 culture-positive biopsies for *S. epidermidis* resulted in U-ITI false-negatives (Table 2). 4 patients suffering from OIAI caused by staphylococci, 3 of which involving *S. aureus*, would have remained undiagnosed utilizing U-ITI alone. These results may be explained by the U-ITI detection limits as the bacteria in these cases were quantified as “sparse growth” or cultivated after broth enrichment (Table 1). The detection limit is reported by the U-ITI manufacturer to be 10^5^ pathogens/mL of *S. aureus* and 10^4^ pathogens/mL for coagulase negative staphylococci (CNS). As *Staphylococcus* species are common causes of orthopedic implant-associated infection [9], improving the detection limit for *Staphylococcus* species in particular would improve the usability of U-ITI. The differentiation of *Staphylococcus lugdunensis* from other CNS would also be preferable, as this bacterium is more virulent and should be interpreted more like *S. aureus* than other CNS [10, 11]. Other studies have reported challenges with detection of microbes in OIAI using the U-ITI [12–15].

In the present study, the U-ITI also identified the pathogen in 6 culture-negative tissue biopsies from 2 patients. Patient 101 was previously positive for *S. pneumoniae* in blood culture and patient 115 was positive for *P. acnes* (now *Cutibacterium acnes*) in 2/5 cultured tissue biopsies. All 5 biopsies were positive for *P. acnes* with the U-ITI system, suggesting that these samples were true positives.

According to international consensus, at least 2 of the 5 biopsies have to be positive for a microbe to be scored as positive [3]. As the Unyvero system can only analyze 2 biopsies at a time, it will take in excess of 13.5 h to diagnose 1 patient. However, if the 2 biopsies analyzed in the first run are positive for the same bacteria, results can be given after ~ 5 h. Of the 15 patients included in our study, 11 were positive for bacteria in all biopsies meaning that the U-ITI system would have the potential to give same day results in 73% of the patients. The median time to detection of pathogen was 47 h [range: 20–168 h] by conventional methods. Hence, the U-ITI system could reduce time to detection of the pathogen considerably.

There are studies investigating the use of synovial and sonication fluid, making it possible to analyze only 1 sample per patient and thereby reducing the time to detection of pathogen to ~ 5 h [12–14, 16–19]. However, limited sensitivity is still an issue, as also concluded in a recent multi-center study [20].

The U-ITI includes a range of antibiotic resistance markers. As median time to phenotypic antibiotic sensitivity test (time to definite antibiotic treatment) was 89 h [range: 44–192 h] by standard methods, the reduction of time can be improved even more using the U-ITI. However, being in an area with a relatively low prevalence of multi-drug resistance, the antibiotic resistance genes included in the U-ITI did not contribute in improving time to correct treatment in the current study. To be beneficial in similar areas inclusion of additional antibiotic resistance genes is warranted. It would improve U-ITI’s utility to include in the panel genes for resistance of important antibiotics in treating orthopedic implant-associated infections such as quinolone and rifampicin resistance genes.

In conclusion, time to detection of pathogens was improved by using the U-ITI. However, the sensitivity of U-ITI compared to conventional cultivation was too low to permit clinical use before the detection limit for *Staphylococcus* species in particular has been optimized. Although in theory, the U-ITI would improve time to correct antibiotic treatment recommendation, it did not reveal the antibiotic resistance prevalent in our samples. Our data do not support use of this assay in diagnostics.

## Limitations

This is a pilot study where the overall number of biopsies was relatively low and culture-negative biopsies were few. There was a limited number of different species detected and most genetic markers for resistance in the Unyvero panel were not relevant to the findings in this study. However, biopsies were collected from 15 consecutive patients reflecting the clinical daily life in low-resistance areas like ours. Due to lack of Unyvero reagents at the time of surgery 22 biopsies were stored at − 80 °C. Storage at − 80 °C may degrade sensitive bacteria and nucleic acid and consequently lower sensitivity of the Unyvero assay. However; Unyvero identifies DNA from both dead and living microbes.

## Supplementary information

**Additional file 1.** Details of criteria fulfilment for orthopaedic implant-associated infection (OIAI) on the included 15 patients based on the criteria described by Parvizi and co-workers [6].

## Data Availability

All data generated or analyzed during this study are included in this published article and its Additional file.

## Abbreviations

CNS: coagulase negative staphylococci
*F. magna*: *Finegoldia magna*
MALDI-TOF: matrix assisted laser desorption ionization time of flight
OIAI: orthopedic implant-associated infections
*P. acnes*: *Propionibacterium acnes* (now *Cutibacterium acnes*)
*S. aureus*: *Staphylococcus aureus*
*S. epidermidis*: *Staphylococcus epidermidis*
*S. pneumoniae*: *Streptococcus pneumoniae*
U-ITI: Unyvero ITI multiplex-PCR application
