# Supplementary material for: Rapid diagnostics of orthopedic implant-associated infections using Unyvero ITI implant and tissue infection application is not optimal for Staphylococcus species identification
Source: BMC Res Notes. 2019 Nov 6;12:725. doi: 10.1186/s13104-019-4755-5 (PMC6836655; doi:10.1186/s13104-019-4755-5)
Supplement: Supplementary file 1 — Additional file 1. Details of criteria fulfilment for orthopaedic implant-associated infection (OIAI) on the included 15 patients based on the criteria described by Parvizi and co-workers [6]. [file 13104_2019_4755_MOESM1_ESM.docx]

**Additional file 1. Details of criteria fulfilment for orthopaedic implant-associated infection (OIAI) on the included 15 patients based on the criteria described by Parvizi and co-workers (6).**

| **ID** | **1. Sinus tract communication** | **2. Culture positive** | **3. Blood/fluids/histology** |
| --- | --- | --- | --- |
| 101 | no* | no | a. CRP  d. purulence |
| 102 | yes | yes | a. ESR, CRP  d. purulence  e. fluid |
| 104 | yes | yes | a. CRP  d. purulence  e. fluid |
| 105 | yes | yes | a. ESR, CRP  d. purulence  e. fluid |
| 107 | no | yes | a. CRP  d. purulence |
| 108 | no | yes | a. ESR,CRP  d. purulence  e. fluid |
| 109 | no | yes | a. ESR, CRP  d. purulence |
| 110 | yes | yes | a. CRP  d. purulence |
| 111 | yes | yes | a. CRP  d. purulence |
| 112 | yes | yes | a. CRP  d. purulence |
| 113 | yes\| | yes | a. CRP  d. purulence  e. fluid |
| 114 | yes | yes | a. ESR, CRP  d. purulence  e. fluid |
| 115 | no | yes | a. ESR, CRP  d. purulence |
| 116 | yes | yes | a. CRP  d. purulence  e. fluid |
| 117 | yes | yes | a. CRP  d. purulence  e. fluid |
| *CT showed abcess  CRP – C-reactive protein  ESR – erythrocyte sedimentation rate | | |  |
